# Evaluation of Patient IgM and IgG Reactivity Against Multiple Antigens for Improvement of Serodiagnostic Testing for Early Lyme Disease

**DOI:** 10.3389/fpubh.2019.00370

**Published:** 2019-12-05

**Authors:** Kevin S. Brandt, Kalanthe Horiuchi, Brad J. Biggerstaff, Robert D. Gilmore

**Affiliations:** Division of Vector Borne Diseases, National Center for Emerging and Zoonotic Infectious Diseases, Centers for Disease Control and Prevention, Fort Collins, CO, United States

**Keywords:** *Borrelia burgdorferi*, Lyme disease, serodiagnostics, multiantigen testing, *in vivo*-expressed antigens

## Abstract

Serologic testing is the standard for laboratory diagnosis and confirmation of Lyme disease. Serodiagnostic assays to detect antibodies against *Borrelia burgdorferi*, the agent of Lyme borreliosis, are used for detection of infection. However, serologic testing within the first month of infection is less sensitive as patients' antibody responses continue to develop. Previously, we screened several *B. burgdorferi in vivo* expressed antigens for candidates that elicit early antibody responses in patients with Stage 1 and 2 Lyme disease. We evaluated patient IgM seroreactivity against 6 antigens and found an increase in sensitivity without compromising specificity when compared to current IgM second-tier immunoblot scoring. In this study, we continued the evaluation using a multi-antigen panel to measure IgM plus IgG seroreactivity in these early Lyme disease patients' serum samples. Using two statistical methods for calculating positivity cutoff values, sensitivity was 70 and 84–87%, for early acute and early convalescent Lyme disease patients, respectively. Specificity was 98–100% for healthy non-endemic control patients, and 96–100% for healthy endemic controls depending on the statistical analysis. We conclude that improved serologic testing for early Lyme disease may be achieved by the addition of multiple borrelial antigens that elicit IgM and IgG antibodies early in infection.

## Introduction

Accurate diagnostic testing for Lyme disease in the early stages of infection is important to deliver proper antibiotic treatment to patients thereby avoiding serious complications that can arise if untreated. Infection by *Borrelia burgdorferi*, the tick-borne bacterial agent of Lyme disease, progresses over three stages: Stage 1; early localized, characterized by a rash (termed erythema migrans) at the tick bite site; Stage 2; early disseminated, characterized by colonization of tissues and organs producing symptoms including myalgia, arthralgia, with acute cardiac or neurologic involvement; and Stage 3; late disseminated, characterized by arthritis and neurological symptoms (1). Antibiotic therapy is effective when administered at all stages, but early treatment following onset of illness represents the best course for successful cure. Based on subjective symptoms similar to several illnesses (e.g., fever, fatigue), clinical diagnoses can be challenging. Patients that exhibit an erythema migrans (EM) rash at the tick bite site and live in regions of endemicity (i.e., habitats where *Ixodes scapularis*, the tick vector for *B. burgdorferi*, resides) are considerations for a correct diagnosis and prompt treatment. *B. burgdorferi* infection does not produce a bacteremia with abundant organisms in the bloodstream, therefore diagnostic testing by culture, microscopic examination, or PCR is not presently feasible. Current laboratory diagnostic tests rely on the detection of anti-*B. burgdorferi* antibodies to indicate patient exposure to this tick-transmitted spirochete, therefore a confirmation of Lyme disease depends on accurate serologic assays that consider the pretest likelihood and thus the predictive value of laboratory tests.

The current serologic testing recommendation from the Centers for Disease Control and Prevention is a two-step approach with the first being an ELISA of a whole cell sonicate or a peptide of *B. burgdorferi*. When this step yields a positive or indeterminate result, the second step consists of the more specific immunoblot (https://www.cdc.gov/lyme/diagnosistesting/labtest/twostep/index.html). Modifications of the first- and second-tier tests that use combinations of whole cell or recombinant borrelial antigens have been cleared by the U.S. Food and Drug Administration and are commercially available for clinical testing (2). However, sensitive serologic testing is limited during the first days, usually <30, after the patient has been subjected to an infected tick bite, as the full antibody repertoire has not developed (3, 4).

Our attempt to improve the sensitivity of serologic assays in patients with early Lyme disease is based on two hypotheses. First, that IgM and IgG antibodies are produced against a set of antigens that are presented by the host's adaptive immune system in the first days following infection. Second, that there are borrelial antigens expressed *in vivo* within the tick or human hosts that are not present in culture-grown whole cell protein lysate, thereby representing targets for early antibodies. Previously, we screened several antigens that were known to be expressed *in vivo* in ticks and mammalian hosts against a panel of Lyme disease patient serum samples and controls (5). The antigens BBA65, BBA70, and BBA73 were selected for IgM serum immunoreactivity evaluation in early Lyme disease patients together with the three antigens currently used in IgM second-tier immunoblotting, OspC, BmpA, and FlaB. We found that a six antigen approach, whereby reactivity against at least 2 of 6 antigens constituted a positive serology, could increase sensitivity without compromising specificity (6). Also in our initial screening of antigens, BBA69 and BBA73 demonstrated IgG reactivity in a set of early Lyme disease patient samples (5).

In this study, we evaluated IgG seroreactivity against the gene products BBA69 and BBA73 together with antigens OspC, DbpA, FlaB, and VlsE in Stage 1 and Stage 2 early Lyme disease patient serum samples, and combined IgM and IgG responses in a multi-antigen approach for sensitivity and specificity determination. We applied two statistical approaches, one of which evaluates all antigens simultaneously and may select different antigen combinations depending on disease category to maximize performance.

## Materials and Methods

### Recombinant Protein Expression and Purification

Truncated (i.e., lacking signal sequence and lipidation motif) genes encoding BBA69, BBA73, OspC, and DbpA were amplified by PCR from *B. burgdorferi* strain B31 genomic DNA using primers described previously (5, 6). Recombinant proteins were generated and purified in soluble form in *Escherichia coli* with the pETite N-His vector following the T7 Expresso system instructions (Lucigen, Middleton, WI). Cloned genes in expression plasmids were transformed into *E. coli* 10G (Lucigen) and selected for growth on Luria-Bertani (LB) medium plates supplemented with 50 ug/ml kanamycin.

Plasmid DNA from transformant colonies was purified by miniprep (Qiagen, Valencia, CA) and was sequenced for insert confirmation. Recombinant plasmids with the correct gene inserts were transformed into *E. coli* BL21(DE3) (Lucigen). Following transformant screening for the appropriate clones, colonies were grown in LB-kanamycin (50 ug/ml) broth, and recombinant protein expression was induced by the addition of isopropyl-D-thiogalactopyranoside (IPTG; 1 mM). Cells were harvested at late-log-phase growth, and recombinant protein was purified under non-denaturing conditions using a nickel-nitrilotriacetic acid (Ni-NTA) Fast Start His tag affinity purification kit (Qiagen). FlaB does not contain a signal sequence, therefore the entire coding sequence was amplified, cloned, and expressed as described (6). The FlaB protein was purified following manufacturer's instructions for preparation of insoluble protein. Proteins were dialyzed into PBS (pH 7.4) and quantified by bicinchoninic acid (BCA) assay (Thermo-Fisher Scientific, Rockford, IL) before use. Purity of recombinant proteins was assessed by SDS-PAGE staining as demonstrated previously (5). Cloning, expression and purification of recombinant VlsE was performed as previously described with the final product dialyzed in PBS (7).

### ELISA

Recombinant antigens were diluted with carbonate buffer (90 mM NaHCO_3_, 60 mM Na_2_CO_3_; pH 9.6) and bound to 96-well Immulon 2HB format plates overnight at 4°C (Thermo Scientific, Rockford, IL) at a final concentration of 200 ng/well. The plate wells were subjected to five washes with Tris-buffered saline–Tween 20 [TBS-T; 20 mM Tris, 140 mM NaCl, 2.7 mM KCl, 0.05% Tween 20 (pH 7.4)] using a BioTek 405 Select plate washer (BioTek, Winooski, VT), followed by addition of blocking buffer (TBS-T with 3% fetal bovine serum) for 45 min at room temperature. Serum samples were diluted 1:100 in blocking buffer, then added to the wells coated with the antigens, and the plates were incubated for 60 min with moderate agitation at room temperature followed by five washes with TBS-T. Alkaline phosphatase-conjugated goat anti-human IgG (H + L, KPL, Gaithersburg, MD) was added at 1:5,000 in TBS, and plates were incubated for 45 min. with agitation at room temperature followed by the wash step. For development, 100 μL of para-nitrophenyl phosphate (PNPP) substrate (Thermo-Fisher Scientific) was added to each well, followed by incubation with agitation at room temperature for 20 min. The reaction was stopped by adding 50 μL of 2 N NaOH to wells. Plates were read at an optical density at 405 nm (OD405) using an ELx808IU Ultra microplate reader (BioTek). Each serum sample was assayed in duplicate. Optimal antigen, serum and conjugate dilutions were determined prior to running the samples as described previously (5). A moderately-reactive serum sample to BBA73 was used as a positive control for each plate, and a low-reactive serum sample to the same antigen was used as a negative control. Optical density data was recorded and used for statistical analysis. Serum sample IgM optical density data was previously performed and recorded as described (6).

### Serum Samples

The Lyme Serum Repository (LSR) was the source of human serum panels used in this study, and samples were collected by the Division of Vector Borne Diseases, Bacterial Diseases Branch, Centers for Disease Control and Prevention. A detailed description of the LSR, which is composed of serum obtained from well-characterized Lyme disease patients, control serum from healthy individuals, and serum from patients with other diseases, has been published (8). Lyme disease patient samples were subdivided into groups as follows: early Lyme disease with EM, which consisted of paired patient serum samples taken at the acute and convalescent phases of disease (stage 1; *n* = 78); early Lyme neuroborreliosis (stage 2; *n* = 9); and early Lyme carditis (stage 2; *n* = 7). Patients with early Lyme disease with EM could be scored as two-tiered negative, but for acceptance into the serum panel, they were required to have well-documented clinical and laboratory (PCR and/or culture) evidence of infection.

This study was carried out in accordance with the recommendations of the Institutional Review Board (IRB) & Research Determinations, Human Studies Team, National Center for Emerging and Zoonotic Infectious Diseases (NCEZID), Centers for Disease Control and Prevention. The protocol was approved by the NCEZID IRB board and determined that it does not include human subjects, as defined under 45 CFR 46.102(f). IRB review was not required. Informed consent and Institutional Review Board approval was granted for the testing of these samples.

### Statistical Analyses and Cutoff Calculations

To normalize for anticipated daily variation of the assay measurements, duplicate positive control wells employing a reactive serum control against rBBA73 were included on each plate. Optical density (OD) values were normalized by dividing all OD values on the plate by the positive plate controls' average OD. Exploratory analysis showed relatively little variance attributable to user or date replications, therefore sample replicates were averaged over these prior to further analysis. Natural logarithms (ln) of the normalized values were computed for use as the primary measure in analyses (data shown in Figure S1).

Upon closer examination of the original data, six of the healthy endemic samples had abnormally high OD values. A follow-up principal components analysis indicated that these samples were indeed outside of the normal range for a typical healthy endemic and so were excluded as controls.

Two methods were used to calculate cutoffs to declare samples positive for *B. burgdorferi* infection. The first method of cutoff determination used a receiver operating characteristic (ROC) curve for each antigen being tested. We selected the cutoff that maximized sensitivity while fixing specificity at 99%. A positive result for the serum sample was declared whenever 2 or more of the 12 antigen measurements (6 antigens for IgM and 6 antigens for IgG) were above their respective cutoffs. For sensitivity of each disease group, we compared the samples to the healthy non-endemic samples. Specificities were calculated by applying the computed antigen cutoffs for the early EM acute group to the healthy and non-Lyme disease samples.

The second method of cutoff determination was to compute a “score” for each sample in a logistic regression model that combined the normalized ln(OD) values for all 12 antigens. For each disease category compared to healthy non-endemic patient samples, we computed the scores by finding the weights for the linear combination (i.e., weighted sum) of normalized ln(OD) values for all antigens that maximized the area under the ROC curve (AUC) (9). Generalized cross-validation (GCV) was used for each of these fits to provide a more robust estimate of the AUC for each linear combination. The linear combinations were computed for each possible subset of antigens (4095 possible sets of the 12 antigens) and ranked by their GCV-AUC values. The top-ranked linear combination was the one with the highest GCV-AUC, and the associated scores from these were then used in ROC analyses to determine the cutoff that maximized sensitivity while fixing specificity at 99%. The cutoff value obtained for all early Lyme disease group samples (i.e., EM acute, EM convalescent, neuroborreliosis, and carditis) was used to determine specificity for the non-Lyme disease and healthy endemic categories combined. We computed 95% confidence intervals (CIs) for sensitivity and specificity when using both methods. The coefficients determine how each antigen OD value is included in overall score of the linear combination. A negative coefficient lowers the score, while a positive coefficient increases it. Because the antigen OD values are scaled within-antigen, the coefficients can also be compared for relative effect, so that, for example, a coefficient of 0.48 is 4 times as impactful as one of 0.12, for the same OD value. When no coefficient listed in the table, its coefficient is 0, meaning that particular antigen does not contribute to the ROC-AUC linear combination for that category.

## Results

### ELISA IgM and IgG Combined Evaluation of Early Lyme Disease Patient Serum Samples Against 12 Antigens

#### Setting Cutoff Values Using Receiver Operator Characteristic (ROC) Curve Analysis

We analyzed the data by setting cutoff values for IgM plus IgG positivity by ROC curve analysis of the healthy non-endemic control serum patient samples vs. each disease group samples. Specificity was set at 99% when determining the ROC cutoff. Table 1 shows the sensitivities for each Lyme patient category with reactivity to ≥2 of the 12 antigens scored as positive.

**Table 1:** IgM plus IgG sensitivity and specificity of early Lyme disease patient samples.

**Patient category**	***N***	**No. positive (% Sensitivity) [95% CI]**
**Lyme disease**		**≥** **2 antigens positive**
Early EM acute	40	28 (70) [55–82]
Early EM convalescent	38	32 (84) [70–93]
Carditis	7	6 (86) [49–99]
Neuroborreliosis	9	8 (89) [57–99]
**Non-Lyme disease**		**No. positive (% Specificity) [95% CI]**
		**<2 antigens positive**
Fibromyalgia	31	1 (97) [84–100]
Mononucleosis	30	4 (87) [70–95]
Multiple sclerosis	21	3 (86) [65–95]
Periodontitis	20	1 (95) [76–100]
Rheumatoid arthritis	21	1 (95) [77–100]
Syphilis	20	8 (60) [39–78]
Healthy endemic	94	4 (96) [90–98]
Healthy non-endemic	102	2 (98) [93–99]

*N, number of samples*.

*ROC cutoffs based on 99% specificity for healthy non-endemic samples*.

Sensitivity for the early EM acute patients was 70% (28/40), with 84% (32/38) of the paired samples representing early EM convalescent testing positive. Sensitivity was 86% (6/7) for the carditis patients, and 89% (8/9) for the neuroborreliosis patients. Although specificity was set at 99% for each individual antigen's ROC cutoff, the specificity for the combined antigen method (<2 positive antigens) for the healthy non-endemic patients was calculated at 98% (100/102) due to the discrete nature of the data. Specificity for the healthy endemic patients, however, was lower at 96% (90/94). The non-Lyme disease patient samples demonstrated a range of specificities from 60 to 97%, with the lowest being syphilis patients (Table 1).

#### Setting Cutoff Values by Linear Combination of Antigen Normalized ln(OD) Values Maximizing the ROC AUC

The second method of deriving cutoffs used the scores calculated from the linear combination of normalized ln(OD) values that maximized the AUC and gave the coefficients (weights) corresponding to the highest GCV-AUC listed for each Lyme disease category (Table 2). Each disease category was compared to healthy non-endemic patient samples. The estimate of the GCV-AUC is shown, as is the positivity cutoff-value determined using the ROC curve derived from the score value for each sample, using the coefficients shown. The estimated sensitivities and 95% CIs using the positivity cutoff given are also shown in Table 2.

**Table 2:** Linear combination giving the highest cross-validated AUC.

**Patient category**	***N***	**No. positive (% Sensitivity) [95% CI]**	**Cutoff value**	**GCV-AUC**	**Coefficients**											
					**IgM**						**IgG**					
					**BmpA**	**FlaB**	**OspC**	**BBA65**	**BBA70**	**BBA73**	**DbpA**	**FlaB**	**OspC**	**VlsE**	**BBA69**	**BBA73**
Early Lyme EM Acute	40	28 (70) [55–82]	0.94	0.97		0.23	−0.18	−0.29	0.09	−0.16	0.11	0.49	0.42	0.61	−0.01	0.02
Early Lyme EM Convalescent	38	33 (87) [73–94]	1.03	0.96	−0.11	0.06	0.14	−0.34		0.03		0.43		0.79	−0.15	−0.03
Carditis	7	7 (100) [65–100]	0.65	1.00	0.10	−0.08	0.54		−0.26		0.56	0.25		0.49		
Neuroborreliosis	9	9 (100) [70–100]	0.08	1.00		0.64	0.61	−0.25	−0.31	0.22					0.06	−0.02
**Non-Lyme disease**		**No. positive (% Specificity** a**) [95% CI]**
Healthy non-endemic	102	1 (99) [65–100]

a *Specificity based on early Lyme EM acute linear combination and cutoff*.

Early EM acute Lyme disease was evaluated and demonstrated that the full subset of antigens (except BmpA) resulted in a GCV-AUC of 0.97, and the corresponding coefficients (weights) for the antigens are shown in Table 2. The positivity cutoff value for the scores computed using these coefficients was 0.94, which resulted in an estimated sensitivity of 70% (28/40) (Table 2). Sensitivity was similarly calculated for early Lyme disease convalescent samples and resulted at 87% (33/38). Sensitivity for neuroborreliosis samples was 100% (7/7), and was 100% (9/9) for carditis samples (Table 2).

Specificities were calculated by comparing all early Lyme disease samples to all non-Lyme disease and healthy endemic samples combined (Table 3). Specificities for the non-Lyme disease samples ranged from 95 to 100% with only 2 false positives; one each in the syphilis and multiple sclerosis groups. Specificity for the healthy endemic patient samples was 100% (Table 3).

**Table 3:** Specificities based on all early Lyme against all non-Lyme diseases and healthy endemics.

					**Coefficients**											
**Patient category**	**N**	**No. positive (% Sensitivity) [95% CI]**	**Cutoff value**	**GCV**	**IgM**						**IgG**					
					**Bmp A**	**FlaB**	**OspC**	**BBA65**	**BBA70**	**BBA73**	**DbpA**	**FlaB**	**OspC**	**VlsE**	**BBA69**	**BBA73**
**All early Lyme**	94	67 (71) [61.4–79.4]	1.21	0.93	−0.14	0.29	0.37		−0.44		−0.04	0.13	0.10	0.72		−0.14
**Non-Lyme disease**	**N**	**No. positive (% Specificity) [95% CI]**
Fibromyalgia	31	0 (100) [89–100]
Mononucleosis	30	0 (100) [89–100]
Multiple sclerosis	21	1 (95) [77–100]
Periodontitis	20	0 (100) [84–100]
Rheumatoid arthritis	21	0 (100) [85–100]
Syphilis	20	1 (95) [76–100]
Healthy endemic	94	0 (100) [96–100]

#### Breakdown of Number of Positive Antigens per Serum Sample Tested

An interesting observation during the ROC analysis of the data was the number of individual serum samples that were positive for at least 3 antigens. As noted in Table 4, several early Lyme disease patients scored positive for 3–7 antigens with some patients showing reactivity to 8–10 antigens. This result indicates how individual patients elicit antibodies early following infection against an array of borrelial antigens. The observation shown in Table 4 indicates the potential to generate improved serological testing utilizing multiple antigens in a combined IgM plus IgG serological assay.

**Table 4:** Number of antigens scored positive per individual serum sample by ROC analysis.

**No. positive antigens^a^**	**0**	**1**	**2**	**3**	**4**	**5**	**6**	**7**	**8**	**9**	**10**	**Sum**
**LYME DISEASE**												
Early Lyme EM Acute	7	5	**7**	**4**	**4**	**4**	**4**	**1**	**1**	**1**	**2**	40
Early Lyme EM Convalescent	4	1	**6**	**2**	**3**	**7**	**5**	**6**	**2**	**1**	**1**	38
Carditis	0	0	**2**	**0**	**1**	**2**	**0**	**0**	**1**	**0**	**1**	7
Neuroborreliosis	0	1	**0**	**1**	**1**	**1**	**1**	**1**	**2**	**1**	**0**	9
**Sum**	11	7	**15**	**7**	**9**	**14**	**10**	**8**	**6**	**3**	**4**	
**NON-LYME DISEASE**												
Fibromyalgia	21	9	**0**	**1**	**0**	**0**	**0**	**0**	**0**	**0**	**0**	31
Mononucleosis	17	9	**3**	**0**	**1**	**0**	**0**	**0**	**0**	**0**	**0**	30
Multiple sclerosis	16	2	**3**	**0**	**0**	**0**	**0**	**0**	**0**	**0**	**0**	21
Periodontitis	16	3	**1**	**0**	**0**	**0**	**0**	**0**	**0**	**0**	**0**	20
Rheumatoid arthritis	16	4	**0**	**1**	**0**	**0**	**0**	**0**	**0**	**0**	**0**	21
Syphilis	5	7	6	2	0	0	0	0	0	0	0	20
Healthy endemic	84	6	3	1	0	0	0	0	0	0	0	94
Healthy non-endemic	94	6	2	0	0	0	0	0	0	0	0	102

a *No Lyme disease patient samples scored positive for 11–12 antigens*.

*The bold numbers signify no. of antigens required for a positive score*.

## Discussion

Several studies have reported on modified serodiagnostic assays for early Lyme disease evolving from standard two-tier testing suggesting a number of approaches to improve sensitivity while maintaining specificity (2, 10–12).

In this study we assessed a multi-antigen strategy to detect IgM and IgG antibody responses in patients with early onset of infection. We hypothesized that antigens synthesized by *B. burgdorferi in vivo* and processed early by the immune system would provide additional targets for detection of the first wave of antibody production. In this study, we combined the 6 antigens described in our previous work for improving IgM serology (BmpA, FlaB, OspC, BBA65, BBA70, and BBA73) with 2 antigens we identified as IgG reactive, BBA69, and BBA73 (5, 6). We also included VlsE, DbpA, FlaB, and OspC for the IgG analysis as these antigens have been documented as seroreactive in patients with early Lyme disease (7, 13, 14). FlaB, OspC, and BBA73 were tested for both IgM and IgG in this study.

We used two statistical approaches to set ELISA cutoff values and calculated sensitivity and specificity based on combined seroreactivity by IgM plus IgG (6 antigens each). In our previous study, we found that the ROC and ROC-AUC provided the most robust computational analyses (6). Consistent with that study, we set cutoffs using the healthy non-endemic patient serum as controls.

With both statistical methods, we found sensitivities of 70 and 84–87% for early acute and early convalescent Lyme disease patients, respectively, using the combined 12 antigen IgM and IgG seroreactivities. These results are increased over the standard 2-tiered testing for these samples at 40 and 61% (8). Our sensitivity results also compare favorably and in some cases are higher than published reports for early Lyme disease detection (10), however it is difficult to compare as these studies used different serum samples than were used here. When the same serum samples were used, our sensitivities were increased over results reported for standard two-tiered tests (STTTs) for both early acute samples (70 vs. 40–48%) and for early convalescent samples (84–87 vs. 61–68%) (2). We also found increased sensitivities over results reported for 2 modified two-tiered tests (MTTTs), i.e., 2-EIA approaches, for early acute samples (70 vs. 48–50%) and early convalescent samples (87 vs. 74–79%) (2). A second study by Pegalajar-Jurado et al., evaluated 3 additional 2-EIA MTTTs against the serum samples used in our study with sensitivities for early acute samples from 50 to 58%, and from 76 to 79% for early convalescent samples, both lower than our results (12).

When calculated by ROC, however, our specificity was lower for healthy endemic serum samples at 96%, with increases in false positives for non-Lyme diseases compared to the STTTs and MTTTs. This result may be reasonably tolerated as clinical diagnoses should differentiate Lyme disease from syphilis and periodontal disease for example. We note that only 1 false positive each for rheumatoid arthritis and fibromyalgia samples were scored, both diseases that could be misdiagnosed as Lyme disease.

Specificities estimated by the more sophisticated ROC-AUC statistical approach resulted in exceptional values of 100% for healthy endemic patients and all non-Lyme patients (except for syphilis and multiple sclerosis which only had one false positive each). Utilization of the ROC-AUC methodology would be useful with an unrestricted, well-studied number of antigens and a sufficiently large set of serum samples where such an algorithm has the potential to maximize the value of the data for sensitivity and specificity by finding the best combination of antigens for each disease category. We showed resultant specificities for the non-Lyme categories based on the cutoff score for all early Lyme categories as an example of the usefulness of this methodology (Table 3).

Combined testing for IgM with IgG resulted in much greater sensitivity than we previously reported for IgM alone. For early acute Lyme samples, sensitivity increased to 70% from 28 to 30% testing with IgM only. For early convalescent Lyme samples, sensitivity increased to 84–87% from 50 to 68% with IgM only (6).

An interesting finding was the number of individual patients in the early stages of infection that reacted positively with 3 or more antigens, and in some cases up to 6–10 antigens. Obviously, although specificity with this number of positives would be nearly 100%, sensitivity would be below an acceptable threshold.

This result suggests that (i) individual patients are unique in their elicitation of antibodies against a spectrum of borrelial antigens, and (ii) infectious Borrelia populations express or harbor a differential array of antigenic proteins which may be amenable to host processing. This finding suggests potential for improved serological testing utilizing multiple antigens in a combined IgM plus IgG serological assay.

In conclusion, several investigations using the multi-antigen approach to improve serologic testing for Lyme disease have been reported underscoring the rationalization for adding antigens for new test algorithms (14–18). A commercial assay would likely employ multiplex testing technology to enhance sensitivity over ELISA formats and provide a platform to screen dozens of antigens (11, 19, 20). These studies and ours represent pilot versions of algorithms for new tests and warrant validation with higher numbers of prospectively and retrospectively collected patient samples.

## Data Availability Statement

All datasets generated for this study are included in the article/Supplementary Material.

## Author Contributions

KB and RG conceived and carried out the experiments. KB, RG, KH, and BB analyzed the data. KH and BB performed the statistical analyses. RG concepted and supervised the study. All authors were involved in writing the paper and had final approval of the submitted and published versions.

### Conflict of Interest

The authors declare that the research was conducted in the absence of any commercial or financial relationships that could be construed as a potential conflict of interest.
