# Supplementary material for: Evaluation of Patient IgM and IgG Reactivity Against Multiple Antigens for Improvement of Serodiagnostic Testing for Early Lyme Disease
Source: Front Public Health. 2019 Dec 5;7:370. doi: 10.3389/fpubh.2019.00370 (PMC6906137; doi:10.3389/fpubh.2019.00370)

**S1 Fig.** The normalized ln(OD) values for the data. Early Lyme samples are in red, healthy samples in black and non-Lyme disease samples in gray.
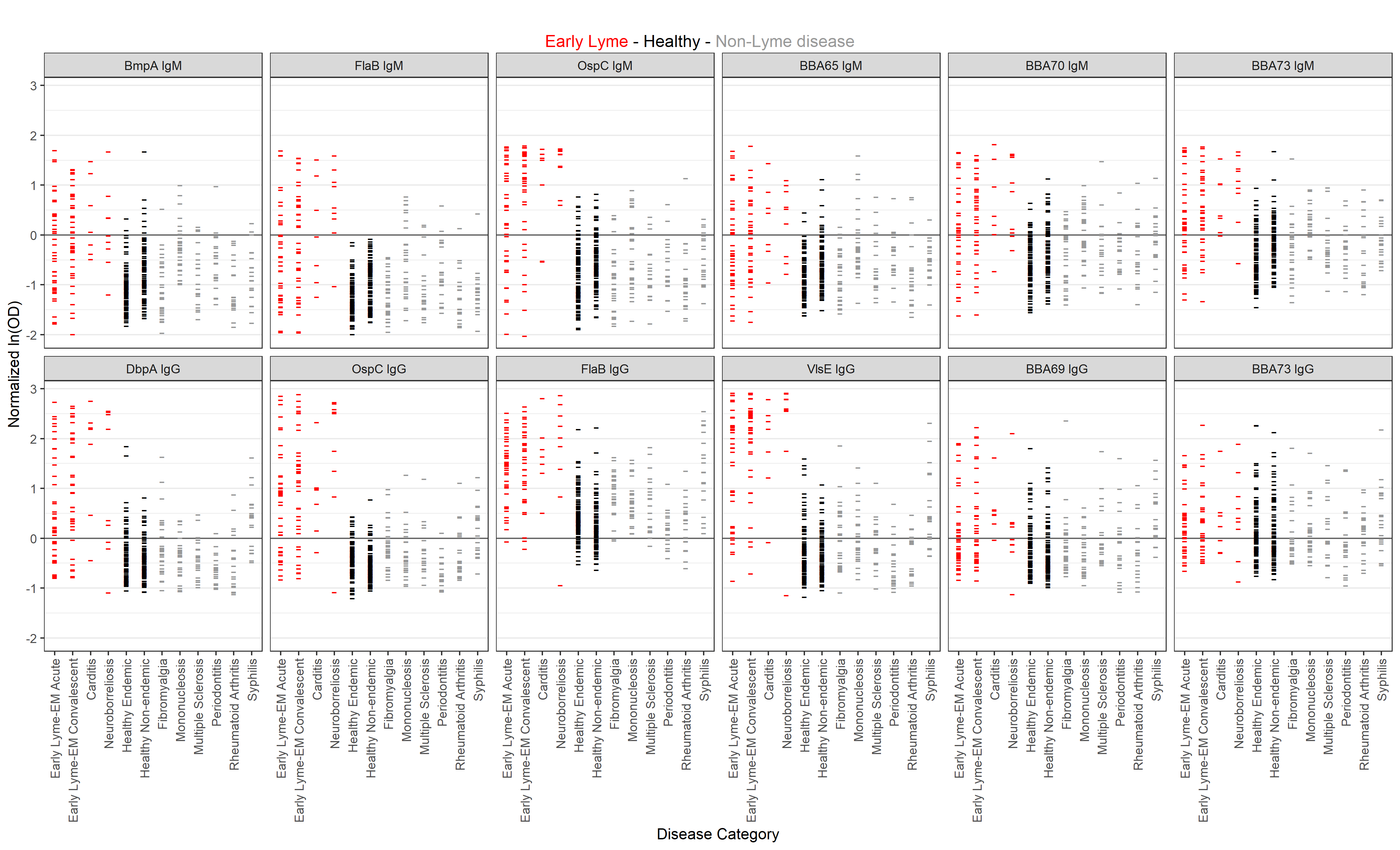

Supplement: Supplementary file 1 [file Data_Sheet_1.docx]
